# Ketogenic Dietary Therapies in Patients with Autism Spectrum Disorder: Facts or Fads? A Scoping Review and a Proposal for a Shared Protocol

**DOI:** 10.3390/nu13062057

**Published:** 2021-06-16

**Authors:** Costanza Varesio, Serena Grumi, Martina Paola Zanaboni, Martina Maria Mensi, Matteo Chiappedi, Ludovica Pasca, Cinzia Ferraris, Anna Tagliabue, Renato Borgatti, Valentina De Giorgis

**Affiliations:** 1 Department of Child Neurology and Psychiatry, IRCCS Mondino Foundation, 27100 Pavia, Italy; costanza.varesio@mondino.it (C.V.); serena.grumi@mondino.it (S.G.); martinapaola.zanaboni@mondino.it (M.P.Z.); martina.mensi@mondino.it (M.M.M.); matteo.chiappedi@mondino.it (M.C.); ludovica.pasca01@universitadipavia.it (L.P.); renato.borgatti@mondino.it (R.B.); 2 Department of Brain and Behavioral Sciences, University of Pavia, 27100 Pavia, Italy; 3 Human Nutrition and Eating Disorder Research Center, Department of Public Health, Experimental and Forensic Medicine University of Pavia, 27100 Pavia, Italy; cinzia.ferraris@unipv.it (C.F.); anna.tagliabue@unipv.it (A.T.)

**Keywords:** ketogenic diet, autism spectrum disorder, scoping review, dietary protocol

## Abstract

Autism spectrum disorder (ASD) is a neurodevelopmental disorder with increasing incidence. An expanding body of literature is examining connections between ASD and dietary interventions. Existing reports suggest a beneficial effect of ketogenic dietary therapies (KDTs) in improving behavioral symptoms in ASD. In this context, the purpose of this scoping review was to identify and map available evidence in the literature about the feasibility and potential efficacy of KDTs in pediatric patients with ASD and to inform clinical practice in the field. Moreover, based on the resulting data from the literature review, we aimed to provide a shared protocol to develop a personalized KDT intervention in patients with ASD. A comprehensive and structured web-based literature search was performed using PubMed and Scopus and it yielded 203 records. Seven papers were finally selected and included in the review. Data were abstracted by independent coders. High variability was identified in study designs and dietary aspects emerged among selected studies. Results supported the effectiveness of KDTs in promoting behavioral improvements. Clinical recommendations on which patients may benefit most from KDTs implementation and difficulties in dietary adherence were discussed.

## 1. Introduction

Autism spectrum disorder (ASD) represents a heterogeneous neurodevelopmental disorder encompassing a complex range of lifelong abnormal social interactions, communication deficits, and repetitive and restricted behaviors [1]. International epidemiological studies report a generalized increase in the prevalence of ASD. A very recent review found 1% to be a conservative median worldwide figure for ASD prevalence [2]. Diagnosis and monitoring are based on clinical criteria and may incorporate standardized scales [1].

Behavioral interventions, directed both at the patient and the “significant others” (i.e., parents, teachers, caregivers), are considered the basic treatment option [3]. Pharmacological treatment options for ASD are limited, and their efficacy and safety have been questioned [4]. There is, therefore, a great need for alternative interventions [5,6]. Several studies have tried to show a possible relationship between dietary habits and autism, starting from the fact that gastrointestinal problems are the most frequent medical comorbidity of ASD [7]. To date, dietary interventions have been mainly based on an “elimination diet,” such as gluten-free and casein-free diets [8], but their level of evidence is limited [9].

Almost 100 years had passed since the first report of the effect of the classic ketogenic diet (CKD) on the emotional–behavioral aspects, when Peterman [10] described improvements in behavior and cognitive functions, concomitant with ketosis, in epileptic patients treated with this dietary regimen, which is a high-fat and low-carbohydrate diet. Since then, the CKD application has become a well-established nonpharmacological treatment [11]. In recent years, increased use of ketogenic dietary therapies (KDTs) has been observed in a wide range of neurological and neuropsychiatric diseases, including ASD [12].

The CKD is a high-fat, adequate-protein, low-carbohydrate diet. Classical protocols are based on a fat-to-carbohydrate ratio of 3 or 4:1 [13]. Adherence to the CKD might be difficult for patients and families. To increase tolerability and palatability, more liberal versions, such as the modified Atkins diet (MAD), the medium-chain triglyceride ketogenic diet (MCTKD), and low glycemic index therapy (LGIT) diet, have been proposed as potential alternatives [11].

Despite several hypotheses, it is still not completely clear how the KDTs and their metabolites may exert therapeutic antiepileptic and neuroprotective effects, thus translating into clinical improvements [14,15].

It is plausible to hypothesize that KDT’s impact on ASD core symptoms may be mediated by its anti-inflammatory effects and its action on mitochondrial function [16]. An alternative explanation involves modulating the microbiota through KDTs as a major factor in producing changes in a patient’s symptoms [17]. In murine models, KDTs resulted in attenuation of some autistic features, suggesting KDTs as a promising treatment option for ASD, despite the limited number of observations [18,19,20,21].

The present review aimed to identify and map available evidence about KDTs potential for pediatric patients with autism and inform clinical practice in the field.

## 2. Materials and Methods

### 2.1. Literature Search

Consistent with the aim, we undertook a scoping review in line with guidelines by Arksey and O’Malley [22] and Peters et al. [23]. Studies that assessed the application and effectiveness of a KDTs protocol for children with ASD were included. In line with this kind of review’s methodological aspects, this study did not focus on a specific study type. Only English-language articles published in peer-reviewed journals were considered.

A comprehensive and structured web-based literature search was performed in December 2020 using PubMed and Scopus to identify primary research articles. In particular, the following keywords were used: (“diet, ketogenic”[MeSH Terms]) AND ((“autism spectrum disorder”[MeSH Terms]) OR (“autistic disorder”[MeSH Terms])); ((TITLE-ABS-KEY (keto)) OR (TITLE-ABS-KEY (ketogenic)) OR (TITLE-ABS-KEY (ketogenic AND diet))) AND ((TITLE-ABS-KEY (autism)) OR (TITLE-ABS-KEY (autism AND spectrum AND disorder)) OR (TITLE-ABS-KEY (autistic AND disorder))).

### 2.2. Study Selection

**Figure 1:**
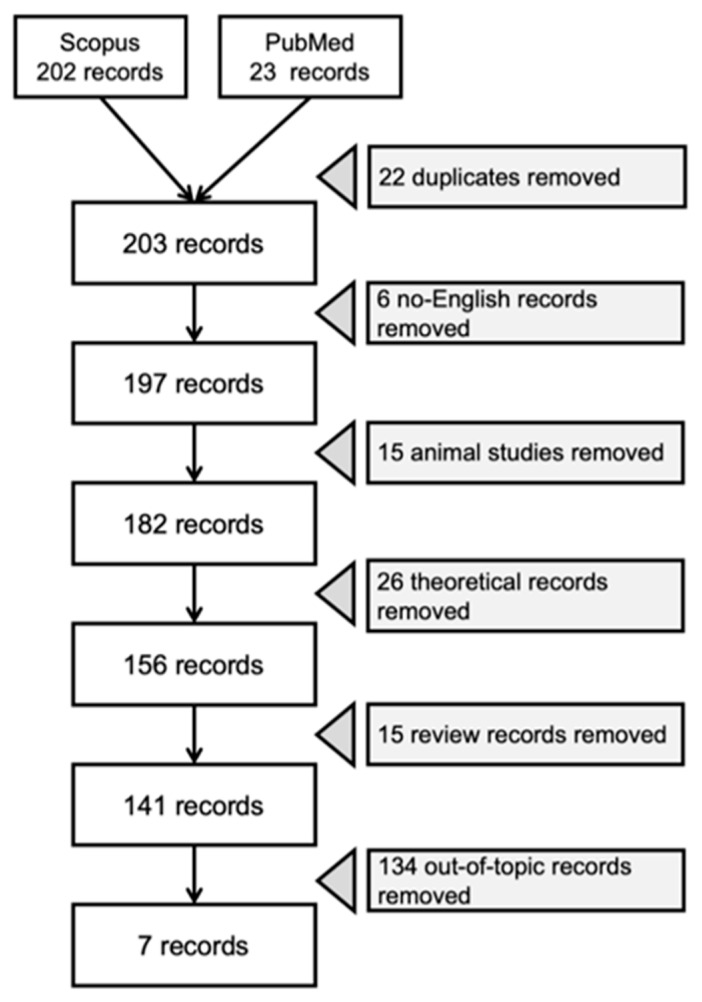
Study selection flowchart.

All studies that investigated the KDTs application and effectiveness for children with ASD were included. Studies identified through the literature search were assessed for inclusion through two stages by two independent authors (CV and SG). First, all records and abstracts were reviewed and analyzed based on the inclusion criteria (language and topic). If abstracts did not include enough information to determine eligibility, articles were selected for further review. Then, the full text of each paper was assessed against eligibility criteria. The study selection flowchart (Figure 1) maps out the step-by-step criteria for screening and the number of records identified, included, and excluded during this process. Out-of-topic records included studies that did not focus on KDTs or the pediatric population diagnosed with ASD.

### 2.3. Data Charting

For each selected study the following data were abstracted: participants’ characteristics (sample size, age, sex, comorbidities), methodological variables (study design, diet characteristics, outcomes measures), and findings of feasibility and effectiveness. Data were analyzed using a qualitative descriptive approach.

## 3. Results

### 3.1. Characteristics of Included Studies

A total of seven studies published between 2003 and 2020 were identified for inclusion. Table 1 summarizes the core characteristics of the selected studies. 

Two of the included records were single case studies [24,25], while the sample size of other studies ranged from 15 to 733, with only one study enrolling more than 50 participants [26]. The age of participants ranged from 2 to 18 years.

Four studies were performed in the USA [24,26,27,28], with one including centers based in Canada [28]. Other studies were performed in Egypt [29], Greece [30], and Poland [25].

Considering the study design, two records adopted an open-label, observer-blinded clinical trial [27,28], two of them were case reports [24,25], while others are categorized as randomized control trial [29], cross-sectional [26], and prospective follow-up studies [30].

Five studies investigated the KDTs effects on both metabolic and behavioral changes, while others focused only on behavioral outcomes [26,30].

### 3.2. Dietary Aspects

#### 3.2.1. Ketogenic Dietary Therapies

As regards the type of diet proposed, the studies show extremely varied dietary protocols. A pure CKD was proposed in only one single report [25], switched after one month of treatment to MAD, and later on LGIT. MAD was also applied in 15 patients recruited in the randomized controlled trial by El-Rashidy et al. [29], followed by 733 patients interviewed by Frye et al. [26]. A variation of the MCT diet, named the John Radcliffe diet, was administered in 30 patients by Evangeliu et al. [30]. In the other three studies, a combined KDTs was proposed. In particular, a KDT gluten-free diet incorporating MCT oil was proposed in 15 and 17 patients by Lee et al. [27] and Mu et al. [28], respectively. A combined gluten-free (GF) and casein-free (CF) KDT (1.5:1 ratio) was administered to a single patient [24].

#### 3.2.2. Periods of Observation

The duration of studies was highly variable, ranging from 3 months to 16 months. In particular, protocols by Lee [27] and Mu [28] provided observations after three months of dietary regimen. Patients enrolled by Evangeliu et al. [30] employed KDT for six months, with continuous administration for four weeks at a time, interrupted by 2-week intervals that were diet-free. More extended periods of observation were reported by Herbert [24] and Żarnowska [25] with 14 and 16, months, respectively.

#### 3.2.3. Ketosis

Ketones levels were reported in four studies [25,27,28,30]. Evangeliou et al. [30] reported levels of β-hydroxybutyrate (BHB) in serum between 1.8 and 2.2 mmol/L during the KDT phase (John Radcliff diet), as compared to levels between 0.8 to 1.5 mmol/L during the diet-free phase. Żarnowska et al. [25] described variations in BHB levels according to different dietary regimens; in particular, during 2:1 CKD, β-hydroxybutyrate level in serum was over 4.0 mmol/L, while it decreased to 2.0 mmol/L during MAD; low to moderate ketones levels were still detectable in urine sample during LGIT.

A significant difference between BHB measures before and after the modified KDT/GF/MCT, with mean levels of 1.351 mmol/L after three months of diet, was reported by Lee et al. [27]. In the same way, Mu et al. [28] demonstrated an increased relative concentration of ketones and metabolites involved in energy metabolism in mitochondria, including 3-hydroxybutyrate, acetoacetate, acetone, and acetylcarnitine.

#### 3.2.4. Laboratory Findings and Changes

Żarnowska et al. [25] reported no clinically meaningful changes in blood laboratory parameters (including those relevant for liver and kidney functions, electrolytes, cholesterol, and lipid profiles, vitamin D, and carnitine) during the observation period. In the same way, stable levels of total cholesterol were reported by Herbert et al. [24]. Conversely, high-density lipoprotein (HDL), low-density lipoprotein (LDL), and significant cholesterol increase were reported by Lee et al. [27] after 3 months of the modified KDT/GF/MCT.

Concerning other laboratory findings, Lee et al. [27] reported a significant decrease in eosinophil blood cell percent.

Mu et al. [28] described different metabolite compositions in ASD post KDT for 3 months, compared to baseline; KDT reduced the relative concentrations of amino acids, including glutamine, tyrosine, phenylalanine, histidine, and alanine; KDT intervention decreased chromium to levels comparable to the control group; KDT treatment for 3 months further increased strontium, antimony, cesium, cerium, and tungsten in ASD.

#### 3.2.5. Side Effects

The side effect profile was reported in three papers [26,27,30], with the percentage of occurrence reported in only one paper [27]. Gastrointestinal side effects included constipation (12.5%) or diarrhea (18.8%) [26,27] and vomiting (18.8%) [27]. Metabolic side effects included weight loss (12.5%), acidosis (6.3%) and hypoglycemia (6.3%). Drowsiness, tiredness, and fatigue were also reported [26]. According to Lee et al. [27], all side effects occurred within the first two to four weeks of diet onset. Conversely, Evangeliu et al. [30] observed no adverse effects usually reported in patients following a KDTs.

#### 3.2.6. Retention Rate

In cohort studies, the retention rate was explicitly reported in four papers [27,28,29,30], with a mean of 65%. In detail, 56% [27], 60% [30], 67% [29], and 78% [28] of patients were able to complete the studies. The reasons for discontinuation were represented by poor compliance [27,29] or intolerance to the dietary regimen, and lack of improvements [30].

### 3.3. Behavioral Outcomes

#### 3.3.1. CARS Scoring

Childhood autism rating scale (CARS) and its updated and advanced versions childhood autism rating scale-2 (CARS-2) are behavioral rating scales designed to provide differential diagnoses from other developmental disorders and assess ASD severity. It is an objective and quantitative tool; rating is based on direct behavioral observation and interview rather than subjective clinical judgment [31].

**Figure 2:**
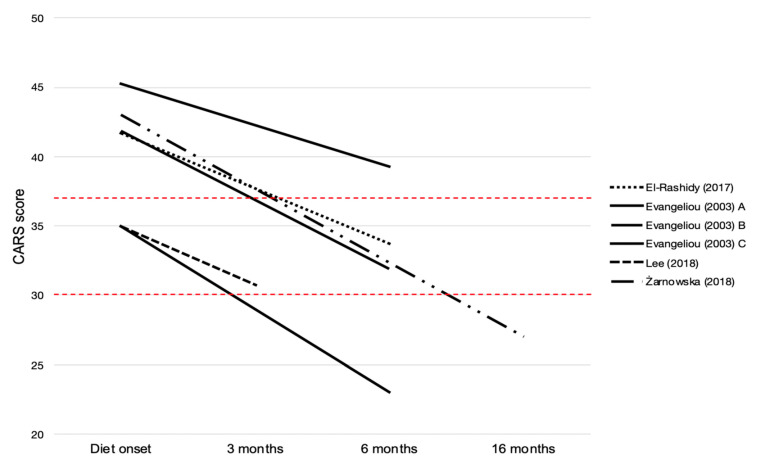
Changes in CARS scoring in reviewed papers. The total scores range from 15 to 60, and the cutoff score to determine autism is 30. A score of <30 is classified as non-autism. A score of 30–36 is classified as mild to moderate autism. A score of ≥37 is classified as severe autism [31].

Changes in CARS scoring were considered a measure of KDTs’ efficacy on autistic symptoms in four studies [25,27,29,30]. As summarized in Figure 2, the total CARS score was reported as decreased in all studies. In particular, in cohort studies, differences between the CARS score before and after KDTs were significantly different, with a mean reduction ranging from 4.25 to 12 points [27,29,30]. Moreover, El-Rashidy et al. [29] reported that patients in the MAD group showed marked improvement in comparison to the GFCF diet group regarding CARS. Additionally, in the single case reported by Żarnowska et al. [25], at the evaluation following KDT, the patient scored 27 points on the CARS scale, with an improvement of 16 points relative to his first evaluation.

Evangeliou et al. [30] proposed a stratification of CARS improvement based on the severity of autistic symptoms presented by patients before the introduction of KDT: patients with mild autistic behavior experienced the most significant improvements.

#### 3.3.2. Other Instruments

Changes at three and six months in autism diagnostic observation schedule-2 (ADOS-2) scores were reported by Lee et al. [27]. After three months on the modified KDT/GF/MCT comparison scores, overall total scores and social affect scores significantly improved, compared to baseline scores, with a mean improvement of 19.9% and 20.7%, respectively. Approximately half (8 of 15) of participants that responded to the modified KDT/GF/MCT improved their overall total ADOS-2 score by at least four points.

Changes in the autism treatment evaluation checklist (ATEC) scores were reported by El-Rashidy et al. [29] after 6 months of dietary regimen. They observed that patients following MAD showed improvements in speech, social, and cognition parameters of ATEC. The MAD group showed marked improvement, in comparison to the CFGF diet group, regarding ATEC scores with a higher percent of changes, with no change in the control group. Lee et al. [27] explored possible associations between biochemical measures and improvement in autistic behaviors. They observed that improvements in AODS-2 scores were significantly associated with HDL and albumin levels from baseline to 3 months on modified KDT/GF/MCT. Moreover, Mu et al. [28] observed that higher responders to KDT depicted the greatest concentrations of 3-hydroxybutyrate and ornithine and lower concentrations of galactose after KDT, compared to lower responders.

The two case reports by Herbert et al. [24] and Żarnowska et al. [25] reported improvement in cognitive function. In the latter case, the cognitive function improvement was quantified through a standardized scale, the Wechsler intelligence scale (WISC-R): full-scale IQ increased from 82 to 99, verbal scale IQ increased from 102 to 113, and performance scale IQ increased from 62 to 83.

Caregivers’ self-reported behavioral changes were registered in four papers [24,25,26,27]. They all described significant emotional and behavioral improvements, as less hyperactivity [24,25,27], less aggressiveness [25], resolution of stereotypies [24]. Social skills were described as improved following KDTs [24,25,27], with greater interest in other people and improved eye contact [27]. Moreover, improvements in language and communication abilities were observed [24,25,27].

## 4. Discussion

Our scoping review aimed to provide a snapshot of the current state of application of KDTs in patients with ASD, considering application protocols and metabolic outcomes, on the one hand, and effects of KDTs on ASD core symptoms, on the other hand.

To date, only seven studies have been focused on the topic. From the methodological point of view, we observed substantial heterogeneity in the study design and duration and applied dietary protocols. Additionally, the assessment of the outcomes (both metabolic and emotional–behavioral) was highly varied, thus making it difficult to systematize the results obtained.

Only one study was designed as a randomized controlled trial, which compared the effect of two different interventions (MAD and GF/CF) versus normal diet [29].

Variability was substantial, even from the point of view of the proposed dietary protocols. CKD with a 2:1 ratio was proposed in only one patient for a single month, subsequently changed into MAD and LGIT later [25]. MAD was the regimen of choice in two studies [26,29], whereas a combination of MAD and MCT was proposed by Lee et al. [27] and Mu et al. [28]. Such great heterogeneity makes it difficult to make comparisons between the different studies. However, it must be recognized that, in studies involving ASD children, who may depict extreme food selectivity [32], it might be necessary to adapt the dietary regimen to increase palatability and tolerability.

Moreover, due to the concomitant application of associated eliminatory diet, i.e., GF, CF [24] the benefits cannot be solely attributed to KD, as it was not the only dietary intervention applied. 

Given the heterogeneity of the KDTs protocols used, the only relevant parameter for understanding the role of KDTs could be the measurement of beta-hydroxybutyrate. Unfortunately, only a few studies reported considerations on ketonemia, and it appeared to be at least discrete [25,27,28].

Mu et al. identified better outcomes in patients with the greatest concentrations of hydroxybutyrate [28], thus indicating better achievement of ketosis. However, this correlation was not identified in other studies [27,29].

The percentage of children who tolerated and adhered to dietary treatment appeared quite satisfactory. However, it is worth noting that such a favorable retention rate is based on basically limited study duration and observation time; therefore, homogeneous studies of longer duration and more extensive case series are necessary to evaluate the effective adherence and tolerability of KDTs.

Side effects were those typically known for KDTs, overall limited in frequency and present in the first weeks after introducing the diet. In the same way, the monitoring of blood tests did not reveal any unexpected alterations. 

Noticeable emotional and behavioral improvements, such as less hyperactive and aggressive behavior, reduced stereotypes, improved social and communication skills, and eye contact, were reported by caregivers’ reports in four out of seven studies [24,25,26,27].

In five out of seven studies [24,25,27,29,30], changes in the scoring of standardized tools were considered a measure of KDTs efficacy on autistic core symptoms. Different rating instruments, such as CARS, ATEC, or ADOS, were used in reviewed studies. Despite this heterogeneity, an improvement in the various scoring was observed, whatever the assessment tool was. In particular, in four out of seven studies, CARS was applied. The validity of CARS has been demonstrated in a variety of studies in children of all ages [33,34]. Significant improvements in CARS scoring were evident in all papers included in our review [25,27,29,30].

Translating our scoping review into clinical recommendations implies a reflection on which patients will benefit most from KDT implementation. Although patient stratification was proposed in a single study [30], it appears that the best responders were patients with mild or moderate autistic symptoms at baseline, while the improvement seemed to appear less relevant for patients with more severe autistic symptoms.

The lack of systematic studies aiming to investigate the effect of KDTs on autistic core symptoms might be due to the extreme caution in suggesting a dietary therapy in a population that could present significant feeding difficulties in terms of dietary intake and eating behaviors (i.e., selective intake, food refusal, food aversion, and atypical eating) [9,32,35], leading to poor tolerance in routine dietary changes. For these reasons, implementing KDTs, similar to any other dietary therapy requiring strict adherence, might be extremely difficult to manage for families, thus increasing parental and familial stress.

## 5. Limitations

As for all scoping reviews, there were limitations to this one. First of all, following guidelines on scoping reviews, an assessment that aimed at formally evaluating the quality of evidence was not performed [36]. Moreover, as scoping review, it gathered information from a wide range of study designs and methods [22,36,37]. As for the review’s results, the literature on this topic is sparse and presents relevant caveats and limitations. High variability was identified in study designs (with the lack of standardized comparison groups in the majority of identified records), patient groups (i.e., age, symptom severity, time of observation), dietary regimen applied, and assessed outcomes. Moreover, the sample size was often small, with most of the studies reporting on less than 20 participants. These limitations and heterogeneity contribute to reduce the specificity and generalizability of the results and highlight the need for a shared and systematized dietary protocol and outcome measures.

## 6. Proposal for a Shared Protocol (Figure 3)

**Figure 3:**
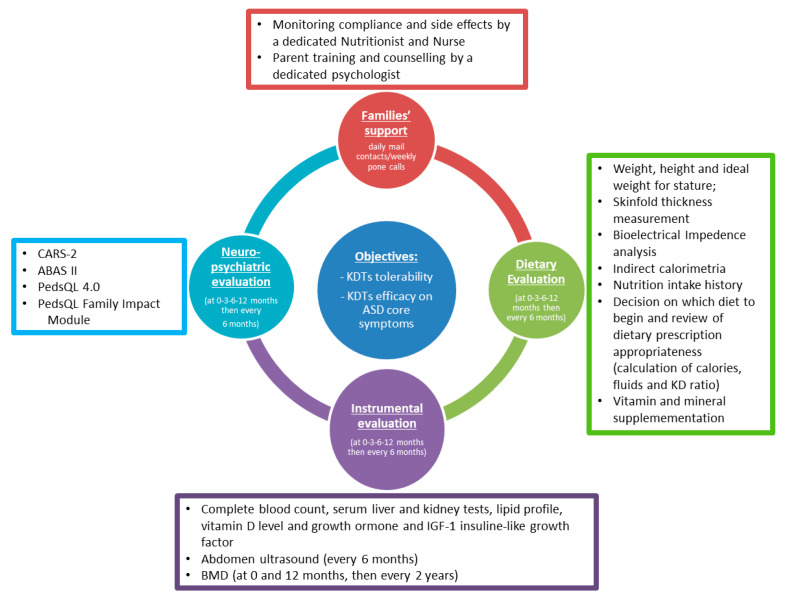
Proposal for a shared KDT protocol in ASD.

To date, no shared protocols are guiding the implementation of KDTs in ASD. Dietary recommendations are based on a single center’s advice and expertise. For this reason, we believe there is a strong need for more standardized protocols for the management of KDTs in the specific subset of patients with ASD, both at the research level and at the level of daily clinical practice (Figure 3).

When proposing a shared protocol, some points should be addressed. Before prescribing such dietary modifications, it is crucial for families to understand clearly the implications of this choice to carry out such a nutritional plan scrupulously and intelligently. In this context, a multidisciplinary approach that includes the cooperation of pediatric neurologists, nutritionists, dietitians, pediatricians and patients’ associations is of great value for providing adequate awareness and social support to families [11]. Based on our experience, careful monitoring of dietary and clinical parameters is essential to support compliance, investigate possible side effects and promptly deal with them [38].

Another point to be determined is identifying which diet will be the most acceptable to children and the easier to be carried out at home, simultaneously achieving satisfactory ketosis levels. Although it is not clear at the moment what the optimal level of ketosis is in these patients, it is conceivable that the ketonemia control may be, at least partly, less stringent than in patients with GLUT1 deficiency syndrome or drug-resistant epilepsy [39], in whom the relationship between the level of ketonemia and control of paroxysmal symptoms is known. However, large-scale studies are needed to clarify this point. We believe a possible strategy for implementing the dietary treatment could be to start with a CKD, with possible changes toward more liberal versions in the event of side effects, poor palatability, and acceptance difficulties.

Based on recommendations on optimal management of KDTs, for an evaluation of KDT tolerability and above all of efficacy, we suggest it should be continued for at least 6 months.

From a nutritional point of view, pre-diet evaluation and counseling, as well as dietary supplementation and monitoring evaluations, should be planned according to the recommendations of the International Ketogenic Diet Study Group [11]. According to the clinical practice currently in use at our center, we suggest laboratory assessment (fully detailed in Table 2) should be performed at baseline and after one, three, and six months from KDT implementation. In patients who prolong KDTs beyond the sixth month period, laboratory assessments should be performed at least twice a year thereafter.

We suggest that autism core symptoms evaluation should be performed through standardized instruments and caregivers’ self-report questionnaires.

CARS-2 is a widely employed and validated tool for assessing ASD symptom burden [33,34]. Based on our review, we believe CARS-2 could be an effective quick, and nimble instrument to monitor changes in AD core symptoms over time. We suggest it could be administered, by an independent, trained rater, before KDT administration and every three months thereafter.

Moreover, Vineland adaptive behavior scale II (VABS II) [34] and parent-rated adaptive behavior assessment system II (ABAS II) questionnaire [34,40] could be feasible, reliable, and accurate measures to quantify meaningful changes in response to treatment [33] and should be administered, in our opinion, by an independent, trained rater, before KD administration, after the review of the treatment at 6 months and then once a year.

Since KDTs are extremely demanding in terms of time and caregivers’ efforts, the patient’s quality of life and family stress related to the diet are essential aspects to monitor. We suggest ad hoc self-administered questionnaires such as Pediatric Quality of Life 4.0 (PedsQoL 4.0) [41,42] and Pediatric Quality of Life Inventory (PedsQL) Family Impact Module [43].

Abbreviations: CARS-2, childhood autism rating scale-2; ABAS-II, adaptive behavior assessment system II; PedsQL 4.0, Pediatric Quality of Life Inventory Version 4.0; KDTs, ketogenic diet therapies; ASD, autism spectrum disorder; KD, ketogenic diet; BMD, bone mineral density.

## Figures and Tables

**Table 1:** Characteristics of included studies. Abbreviations: Y, year; NA, not available; ADHD, attention-deficit/hyperactivity disorder; RCT, randomized controlled trial; MAD, modified Atkins diet; MCT, medium-chain triglyceride; CKD, classic ketogenic diet; LGIT, low-glycemic index therapy; CARS, childhood autism rating scale; ATEC, autism treatment evaluation checklist; ADOS, autism diagnostic observation schedule.

Study	Country	Study Design	Comorbidity	Sample Size	Females (%)	Mean Age (Y) (Range)	Ketogenic Dietary Therapies	Laboratory Outcomes	Behavioral Outcomes
El-Rashidy et al., 2017	Egypt	RCT	NA	15	26.7	5.29 (3–8)	MAD with approximately 60% of the calories from fat sources, 30% from proteins, and 10% from carbohydrates	Complete blood count, serum electrolytes, carnitine, and β-hydroxybutyric acid	Changes in CARS and ATECafter 6 months
Evangeliou et al., 2003	Greece	Prospective follow-up	NA	30	46.6	7 (4–10)	John Radcliffe diet: 30% of energy as medium-chain triglyceride oil, 30% as fresh cream, 11% as saturated fat, 19% as carbohydrates, and 10% as protein.	NA	Changes in CARS after 6 months
Frye et al., 2011	USA	Cross-sectional	Clinical seizures, subclinical epileptiform discharges	733	33	12	MAD	NA	Perceived effect on seizures, sleep, language, verbal and non-verbal communication, stereotyped/repetitive movements, rigidity, hyperactivity, attention, and mood.
Herbert & Buckley, 2013	USA	Case report	Epilepsy	1	100	12	Gluten-free casein-free ketogenic 1.5:1 ratio	Cholesterol	Clinical improvements after 14 months
Lee et al., 2018	USA	Open-label, observer-blinded clinical trial	NA	15	13.3	3.3 (3–13)	Modified ketogenic gluten-free diet regimen with supplemental MCT	High-density and Low-density lipoprotein; Cholesterol; Eosinophil blood cell percent	Changes in CARS after 3 months and changes in ADOS after 3 and 6 months
Mu et al., 2020	USA and Canada	Open-label, observer-blinded clinical trial	NA	17	11.76	9 (2–17)	Modified KD regimen consisted of a gluten-free diet incorporating MCT oil	Metabolic changes	Changes in ADOS after 3 months
Żarnowska et al., 2018	Poland	Case report	ADHD	1	0	6	CKD with a 2:1 ratio. After 1 month on this classic KD, the diet was switched per the parents’ request to a MAD. After five months on the MAD, patient was placed on the LGIT.	Blood parameters	Changes in CARS after 16 months

**Table 2:** Laboratory assessment currently in use in our center for patients under KDTs.

*Laboratory Assessment*
Capillary Ketonemia *
**Complete blood count with platelets**
**Serum Electrolytes**
Sodium
Potassium
Chlorine
Calcium
Phosphorus
Magnesium
Zinc
Selenium
**Serum Metabolic parameters**
Blood glucose
Triacylglycerols **
Total cholesterol **
Low-density lipoprotein cholesterol **
High-density lipoprotein cholesterol **
Total lipids **
Uric acid ***
**Serum parameters of nutritional status**
Total protein concentration
Prealbumin
Albumin
**Serum Liver and Kidney profile**
AST
ALT
Blood urea nitrogen
Creatinine
Total bilirubin
Gamma-GT
Pseudocholinesterase
**Serum Vitamins**
Folic Acid
Vitamin B12
25-hydroxyvitamin D
**Serum hormonal profile**
Insulin ****
IGF1 ***
Growth hormone ****
**Serum Iron profile**
Iron
Ferritin
Transferrin

* Home monitoring should be performed twice a day during the KDTs induction phase; subsequent monitoring will be provided during follow-up at referral center. ** Dyslipidemia might be a common adverse effect; however, it mitigates in a short time by KDT adjustments and providing nutritional integration. *** Transient hyperuricemia might be an early transient adverse effect. **** Growth retardation may occur in a minority of children treated with KDTs.
